# General Anesthesia With Successful Immediate Post-operative Extubation for Sarcoma Excision in a 61-Year-Old Male With Kennedy’s Disease

**DOI:** 10.7759/cureus.21956

**Published:** 2022-02-06

**Authors:** Raymond Evans, Allan R Escher, Daniel A Nahrwold, Jamie P Hoffman

**Affiliations:** 1 Anesthesiology, Moffitt Cancer Center and Research Institute, Tampa, USA; 2 Anesthesiology/Pain Medicine, Moffitt Cancer Center and Research Institute, Tampa, USA

**Keywords:** poly-q disease, anterior horn cell disease, motor neuron disease, x-linked recessive spinal and bulbar muscular atrophy, bulbospinal neuronopathy, extubation response, glottic edema, x-linked genetic diseases, spinobulbar muscular atrophy, kennedy's disease

## Abstract

Kennedy’s disease (KD), also known as spinal and bulbar muscular atrophy (SBMA), is a rare, X-linked recessive androgen receptor gene mutation affecting approximately one in 40,000 males. A prominent anesthetic concern in patients with KD is their ability to maintain a patent airway following general anesthesia. We present the case of a 61-year-old man with a history of KD presenting for a left thigh sarcoma excision. The patient received a general anesthetic with endotracheal tube placement, was extubated in the operating room upon completion of the surgery, and had an uneventful post-operative course.

## Introduction

Kennedy's disease (KD) or spinal and bulbar muscular atrophy (SBMA) is an atypical, X-linked hereditary disease, affecting the lower motor neurons; it causes gradual muscular weakness with a largely normal life expectancy [1]. In 1968, William R. Kennedy first elucidated the hereditary nature of the disease - it was later identified as a rare trinucleotide repeat expansion disorder encoding glutamine [1]. The relatively few case reports involving patients with KD undergoing general anesthesia all underscore the importance of a careful airway assessment prior to extubation [2]. The dysfunctional bulbar musculature and attendant predisposition to laryngospasm represent daunting challenges to confront during emergence [3]. The impact on the lingual complex may manifest as obstructive sleep apnea (OSA), dysphagia, and dysgeusia [4]. Moreover, the observed relationship between general endotracheal anesthesia and subsequent glottic edema in patients with advanced disease presents a significant anesthetic concern and suggests a possible link between the severity of disease and post-operative outcome [5].

Little is known about how predictably patients with KD can be neuromuscularly reversed from blockade with conventional acetylcholinesterase inhibitors. Furthermore, there is a paucity in the literature of patients with KD undergoing general anesthesia. There is only one reported use of the reversal agent sugammadex [2]. Additionally, post-operative outcomes such as dysphagia, tracheostomy with prolonged mechanical ventilation, and tension pneumothorax have been reported [5].

## Case presentation

A 61-year-old male, American Society of Anesthesiologists (ASA) Physical Status (PS) III, presented for a left thigh sarcoma excision. His past medical history was significant for KD, myxoid liposarcoma of the left thigh, hyperlipidemia, chronic back pain, lower extremity neuropathy, and restless leg syndrome. The patient stated he could no longer climb stairs due to hip pain. There were no complaints of fatigue, dysphagia, or difficulty with clearance of oral secretions. Additionally, he denied dysphonia, dyspnea, and muscle spasm. However, the patient did report a history of paroxysmal globus pharyngeus. These episodes lasted for several minutes and had increased in frequency prior to surgery. On physical exam, the patient had a regular heart rate and rhythm, and clear breath sounds bilateral. Examination of the neck revealed a midline thyroid and no masses were appreciated. However, the patient was noted to have recurrent fasciculations of the perioral muscles during the pre-anesthetic assessment and had 5/5 strength in bilateral upper and lower extremities.

The surgery was scheduled for 2.5 hours and the anticipated blood loss was under 50mL. The patient had no previous anesthetic exposure. A lumbar epidural with monitored anesthesia care (MAC) was chosen over a subarachnoid block in order to provide gradual control over the block level. Optimal pain control with minimal IV and inhaled anesthetic exposure, decreased narcotic consumption, a lower risk for post-operative nausea and vomiting (PONV), and increased patient satisfaction were some of the reasons for choosing this anesthetic technique. Additionally, avoiding airway instrumentation and manipulation, the possible need for neuromuscular blockers when intubating, and any potential airway sequelae associated with general anesthetics in patients with KD - all played significant roles in our anesthetic plan.

The patient was sedated in the preop area with 1mg of midazolam and 50 mcg of fentanyl. He was placed in the sitting position and a lumbar epidural catheter was placed in a sterile fashion at lumbar (L) level L4-5 using a midline approach on the first attempt. Loss of resistance (LOR) was with air, aspiration was negative, and test dose was negative using 3mL of 1.5% Lidocaine with epinephrine (1:200,000). LOR to air occurred at 7cm, and the catheter was threaded to 12cm and secured with a large transparent dressing.

The patient was transported to the operating room (OR) and connected to standard ASA monitors. The epidural was incrementally dosed with 5mL of a mixture containing 0.2% ropivacaine, 5mcg/cc fentanyl, and roughly 8mg/mL sodium bicarbonate. After receiving 25mL, the patient developed a sensory level to thoracic (T) level T8 bilaterally as confirmed by loss of temperature sensation. The entire right lower extremity was insensate to pinprick and hemostat pinch, but the left lower extremity sensory blockade remained incomplete, and the patient reported discomfort with the hemostat pinch. One cause of a unilateral blockade can be anatomical such as the presence of a plica mediana dorsalis, a connective tissue in the epidural space. More commonly, the epidural catheter may be withdrawn slightly and an additional bolus may be given. However, to avoid the possibility of a higher thoracic level sensory blockade, the decision was made to proceed with general anesthesia. Induction occurred with 150mg propofol and 100mcg of fentanyl. Once spontaneous ventilation ceased, a 7.0mm cuffed endotracheal tube was placed via a Miller 2 blade with a grade 2 glottic view. The placement was confirmed with bilateral breath sounds and positive end-tidal CO2 (EtCO2). The use of a supraglottic airway was ruled out to minimize the risk of aspiration. Maintenance with inhalational anesthesia ensued with desflurane and the epidural catheter infusion of said mixture continued at 5mL/hr. The operation lasted 3.5 hours. The estimated blood loss (EBL) was 50mL, urine output was 275mL, crystalloid intake was 2.5L, and no additional opiates or muscle relaxants were used. The patient remained on pressure support ventilation for the last 1.5 hours. of the case, and upon completion was extubated without complication. The patient arrived in the post-anesthesia care unit (PACU) comfortable, awake, and conversant, and was discharged to his floor bed within an hour. The remainder of the hospital course was uneventful with effective patient-controlled epidural anesthesia (PCEA) use for pain control until post-operative day (POD) 2, at which point the patient was discharged home.

## Discussion

The primary challenges confronting anesthesiologists when caring for patients with KD include a weak bulbar musculature that is prone to laryngospasm, proximal lower limb muscle atrophy or immobility, and a propensity to aspirate [1-5]. It is for this reason that we chose epidural anesthesia with MAC as the primary surgical anesthetic. The operative location, a relatively short, expected case duration, and low anticipated blood loss made it possible to avoid general anesthesia with mechanical ventilation. It provides the advantage of optimal pain control intraoperatively and post-operatively, while simultaneously decreasing the likelihood of invasive airway instrumentation and subsequent management. Furthermore, the safe and effective use of epidural anesthesia in patients with lower motor neuron disease, including KD, has been demonstrated in multiple reports [6-8].

The decision to switch to a general anesthetic was made after a more than reasonable attempt was undertaken at dosing the lumbar epidural catheter. After injection of 15mL, the catheter was withdrawn 2cm and secured at 10cm before dosing with the remainder of the local anesthetic mixture. The risk of proceeding with general anesthesia in this highly functional patient was weighed against the risk of proceeding with alternative forms of regional anesthesia (e.g., spinal, femoral, and sciatic nerve blockade, additional epidural attempts), and it was decided that general anesthesia was a relatively safe and reasonable option for this patient.

The decision to induce without neuromuscular blockade was primarily due to the desire to avoid exacerbations to an already weak lower limb-girdle and bulbar musculature, and the potential for airway compromise or aspiration post-operatively [9-10]. The nature and location of this surgery allowed for a general anesthetic without the use of neuromuscular blockade, and thereby reduced the possibility of post-operative weakness from the residual blockade.

Successful extubation in the immediate post-operative setting was facilitated by multiple factors. Perhaps most importantly, the patient’s preoperative functional status was limited but relatively good. He was able to ambulate without assistance for flat walking of roughly 100m but required assistance for further distances, or those with an incline. His phonation was essentially normal with mild fasciculations in the perioral musculature. The lack of comorbidities and relatively young age also contributed to this successful extubating. The possible need for post-operative mechanical ventilation should certainly be discussed with patients with KD presenting for surgery. However, immediate post-operative extubation can be considered a safe option in patients with a relatively functional preoperative status undergoing short and uncomplicated surgery.

## Conclusions

The anesthetic management of patients with KD is both a challenge and an opportunity. A frank discussion with the patient regarding the possibility of mechanical ventilation after surgery is prudent. In appropriate cases, the use of regional anesthesia, including neuraxial and peripheral techniques can mitigate the airway risks of general anesthesia. In the case presented, an initial plan for regional anesthesia was transitioned to general anesthesia. The successful immediate post-operative extubation of this patient was accomplished without the use of muscle relaxants or reversal agents.

## References

[1] Breza M, Koutsis G (2019). Kennedy's disease (spinal and bulbar muscular atrophy): a clinically oriented review of a rare disease. J Neurol.

[2] Takeuchi R, Hoshijima H, Doi K, Nagasaka H (2014). The use of sugammadex in a patient with Kennedy's disease under general anesthesia. Saudi J Anaesth.

[3] Sperfeld AD, Hanemann CO, Ludolph AC, Kassubek J (2005). Laryngospasm: an underdiagnosed symptom of X-linked spinobulbar muscular atrophy. Neurology.

[4] Bordoni B, Escher AR (2021). A missing voice: the lingual complex and osteopathic manual medicine in the context of five osteopathic models. Cureus.

[5] Niesen AD, Sprung J, Prakash YS, Watson JC, Weingarten TN (2009). Case series: anesthetic management of patients with spinal and bulbar muscular atrophy (Kennedy's disease). Can J Anaesth.

[6] Okamoto E, Nitahara K, Yasumoto M, Higa K (2004). Use of epidural anaesthesia for surgery in a patient with Kennedy's disease. Br J Anaesth.

[7] Kochi T, Oka T, Mizuguchi T (1989). Epidural anesthesia for patients with amyotrophic lateral sclerosis. Anesth Analg.

[8] Hara K, Sakura S, Saito Y, Maeda M, Kosaka Y (1996). Epidural anesthesia and pulmonary function in a patient with amyotrophic lateral sclerosis. Anesth Analg.

[9] Finsterer J (2010). Perspectives of Kennedy's disease. J Neurol Sci.

[10] Amato AA, Prior TW, Barohn RJ, Snyder P, Papp A, Mendell JR (1993). Kennedy's disease: a clinicopathologic correlation with mutations in the androgen receptor gene. Neurology.

